# Evolution of the Gut Microbiota and Its Fermentation Characteristics of Ningxiang Pigs at the Young Stage

**DOI:** 10.3390/ani11030638

**Published:** 2021-02-27

**Authors:** Hao Li, Longteng Ma, Zhiqing Li, Jie Yin, Bie Tan, Jiashun Chen, Qian Jiang, Xiaokang Ma

**Affiliations:** College of Animal Science and Technology, Hunan Agricultural University, Changsha 410128, China; LLH0920@163.com (H.L.); malongteng2021@163.com (L.M.); maxiaokang8888@163.com (Z.L.); yinjie2014@126.com (J.Y.); bietan0412@hotmail.com (B.T.); jschen@hunau.edu.cn (J.C.); jiangqianisa@gmail.com (Q.J.)

**Keywords:** Ningxiang pigs, gut microbiota, evolution, fiber-degrading bacteria, short chain fatty acids

## Abstract

**Simple Summary:**

The current study described the evolution of the gut microbiota of an indigenous pig breeds, Ningxiang pigs (NXP), from one week before weaning to the end of nursery. The results showed that dietary factors mainly drove the evolution of the microbial community of NXP. Our results contributed to a better understanding of the evolutionary characteristics and influencing factors of the gut microbiota of indigenous pig breeds.

**Abstract:**

The current study aimed to investigate the evolution of gut microbiota and its influencing factors for NXP in youth. The results showed that Shannon index increased from d 21 to d 28 whereas the ACE index increased from d 21 until d 60. Firmicutes, mainly Lactobacillus dominated on d 21. The Bacteroides and Spirochetes showed highest relative abundance on d 28. Fiber-degrading bacteria, mainly *Prevotellaceae*, *Lachnospiraceae*, *Ruminococcaceae*, *Muribaculaceae*, and *Oscillospiraceae_UCG−002*, dominated the microbial communities at d 28 and d 35. The microbial communities at d 60 and d 75 contained more *Clostridium_sensu_stricto_1*, *Terrisporobacter* and *Oscillospiraceae_UCG−005* than other ages, which had significantly positive correlations with acetate and total SCFAs concentration. In conclusion, the evolution of gut microbiota was mainly adapted to the change of dietary factors during NXP growth. The response of fiber-degrading bacteria at different stages may help NXP better adapt to plant-derived feeds.

## 1. Introduction

There are inextricable relationships between a gut microbiota and its host, such as nutrient digestion and absorption, gastrointestinal development and health, energy metabolism and immunologic functions [1]. Thus, the gut microbiota is called the “second genomes” of animals [2]. From birth to death, the gut microbiota of mammals undergoes a dynamic process where its structure and function evolves with the mutations from ages, diets, and environment [3,4,5].

The gut microbes of pigs are similar to those of humans, mainly Firmicutes and Bacteroidetes, and contain 500 to 1000 species [6,7]. Anaerobic bacteria account for more than 99% whereas aerobic bacteria only account for about 1% of total microbial communities [7]. Previous studies have described the evolution of the gut microbiota of some cultivated pig breeds under specific settings and feed regimens [8,9,10,11]. However, it is not well understood about the characteristics of gut microbiota in those indigenous pig breeds, especially because the evolution of their microbial community has not been reported yet. Ningxiang pig (NXP), an indigenous breed from Hunan Province, China, still retains many excellent traits, including dietary fiber tolerance, premium pork, disease resistance and antistress ability [12,13]. Short chain fatty acids (SCFAs) are the metabolites of gut microbiota and have been reported to be potentially related to these traits in some model animals [14,15].

Therefore, the current study was aimed to investigate the influence of age, diet and housing factors in the gut microbiota and fecal SCFAs composition of NXP in youth under commercial feeding conditions. More information could be provided to understand the evolution of gut microbiota and its influencing factors on indigenous pig breeds.

## 2. Materials and Methods

The animal handling and all procedures of this study have received approval from the Animal Care and Use Ethics Committee of the Hunan Agricultural University (Changsha, China).

### 2.1. Animals, Diet, and Housing

**Figure 1:**
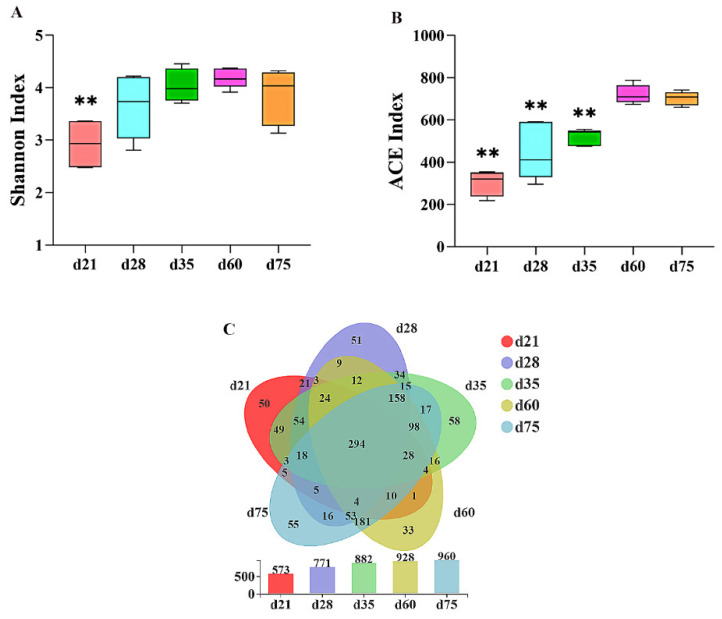
The Shannon index (**A**) and ACE index (**B**), and OTUs (**C**) of fecal microbes. “**” represent significant difference, *p <* 0.05.

The animal feeding was carried out in a commercial pig farm. Eight healthy NXP (female) from eight litters with an initial weight of 5.02 ± 0.19 kg were marked with ear tags at 14 days of age. After a week of adaptation, these piglets experienced different dietary and housing factors as shown in Figure 1. The dietary factors were as follows: mixed intake stage around d 21 main breast milk intake and supplemented by suckling pig feed from d 21 to d 28 of age (MSPF); weaning and feeding only suckling pig feed on d 28 of age (Weaning); suckling pig feed from d 29 to d 35 of age (SPF); nursery pig feed from d 36 to d 75 of age (NPF). The housetransfer was performed on d 29 from the lactation house (LH) to the nursery house (NH) and on d 61 from the NH to the growing–finishing house (GFH). All the diets came from this commercial farm and the chemical composition of breast milk and feeds was shown at Table 1 and the formulas were shown in Supplementary Table S1. During the whole period, the temperature of all stalls is maintained at 25 to 28 ℃ and the relative humidity was between 55% and 75%. All the pigs were provided ad libitum access to water and diets.

### 2.2. Sample Collection

One hundred gram feed samples of each diet at different stages were collected and stored at −20 °C for chemical analysis. Fresh fecal samples on d 21 (*n* = 8), d 28 (*n* = 8), d 35 (*n* = 8), d 60 (*n* = 8) and d 75 (*n* = 8) were collected, divided into two portions (1.0 g~1.5 mL) in centrifuge tubes and stored at −80 °C after quick-freezing in liquid nitrogen. Weight information of pigs at the time of sampling was shown in Supplementary Table S2. After cleaning the udder and abdomen of the sows and disinfecting them with potassium bisulfate complex solution, the milk was manually squeezed at 15 mL per sow and stored at −20 °C.

### 2.3. Chemical Analysis

Dry matter (DM, AOAC 930.15), ash (methods 942.15), crude protein (CP, methods 990.03) and ether extract (EE, method 920.39) contents were determined following the AOAC (2006) procedures. According to the methods of AOAC (2007), soluble dietary fiber (SDF), insoluble dietary fiber (IDF) and total dietary fiber (TDF) were analyzed by using the Ankom Dietary Fiber Analyzer (Ankom Technology, Macedon, NY, USA). Neutral detergent fiber (NDF), acid detergent fiber (ADF) and acid detergent lignin (ADL) were detected using the filter bags (Model F57; Ankom Technology) and fiber analyzer equipment (ANKOM200 Fiber Analyzer, Ankom Technology). The milk samples were separately analyzed for the concentrations of fat, protein, lactose, and DM using a Milko-Scan FT 120 (Foss Electric, Hillerford, Denmark).

### 2.4. Fecal SCFAs Analysis

The concentrations of SCFAs including acetate, propionate, butyrate and valerate were analyzed using the gas chromatographic method. Briefly, approximately 1.0 g of feces were first homogenized in the 1.5 mL deionized water. After centrifuged at 15,000× *g* for 10 min at 4 °C, supernatants (1 mL of each) were acidified with 25% metaphosphoric acid at a ratio of 1:5 (1 volume of acid for 5 volumes of sample) for 30 min on ice. The sample was injected into a GC 2010 series gas chromatograph (Shimadzu, Japan) equipped with a CP-Wax 52 CB column 30.0 m × 0.53 mm i.d (Chrompack, Netherlands). The injector and detector temperatures were 75 °C and 280 °C, respectively. All procedures in GC were performed in triplicate.

### 2.5. Microbiota Analysis

Total genomic DNA were extracted by using a Stool DNA Isolation Kit (Tiangen Biotech Co., Ltd., Beijing, China) following the manufacturer’s instructions. The quantity and quality of extracted DNAs were measured with a NanoDrop ND−1000 spectrophotometer (Thermo Fisher Scientific, Waltham, MA, USA) and agarose gel electrophoresis, respectively. The genes of bacteria 16S ribosomal RNA in the region of V3-V4 were amplified by using polymerase chain reaction (PCR) with primers (515F 5′-barcode- GTGCCAGCMGCCGCGG)-3′ and 907R 5′-120CCGTCAATTCMTTTRAGTTT-3′). Electrophoresis was applied to analyze the integrity of PCR amplicons with the Tapestation Instruction (Agilent technologies, Santa Clara, CA, USA). AxyPrep DNA Gel 122Extraction Kit was chosen to extract and purify PCR amplicons by using 2% agarose gels (Axygen 123Biosciences, Union City, CA, USA) then the production was quantified using QuantiFluor™ -ST and sequenced on the Illumina MiSeq system. QIIME software was used to demultiplex and quality-filtered raw Illumina fastq files. Operational taxonomic units (OTUs) were defined as a similarity threshold of 0.97 using UPARSE. Then UCHIME was applied to identify and delete the unnormal gene sequences. Ribosomal Database Project (RDP) database (http://rdp.cme.msu.edu/ (accessed on 15 January 2021)) was also referenced to take the taxonomy-based analysis for OTUs using RDP classifier at a 90% confidence level.

### 2.6. Bioinformatics Analysis

OTUs representing <0.005% of the population were removed, and taxonomy was assigned using the RDP classifier. The relative abundance of each OTU was counted at different taxonomic levels. Then, bioinformatics analysis was mainly performed using QIIME (v1.7.0) and R packages (v3.2.0). The OTU table in QIIME was used to calculate OTU-level alpha diversity indices, while β-diversity was assessed by principal coordinate analysis (PCoA) and cluster analysis.

### 2.7. Statistical Analysis

SCFAs concentrations and the relative abundance of microbiota were analyzed by the Repeated Measure Procedure in General Linear Model of SPSS 21.0 (SPSS Inc., Chicago, IL, USA). Spearman correlation between bacteria and SCFAs was performed by R. For all tests, *p* < 0.05 was considered as significant difference.

## 3. Results

### 3.1. Biodiversity Changes of the Microbial Community

The OTUs of the fecal samples from NXP at ages of d 21, d 28, d 35, d 60 and d 75 were 573, 771, 882, 928 and 960, respectively, wherein 294 common OTUs were identified (Figure 1C). The α-diversity of microbiota including Shannon index and ACE index were presented in Figure 1A and 1B, respectively. Fecal samples from d 21 had lower Shannon index than those from other ages (*p* < 0.01), while fecal samples from d 21, d 28 and d 35 had lower ACE index than those from d 75. However, there was no significant difference in α-diversity indexes between d 60 and d 75.

**Figure 2:**
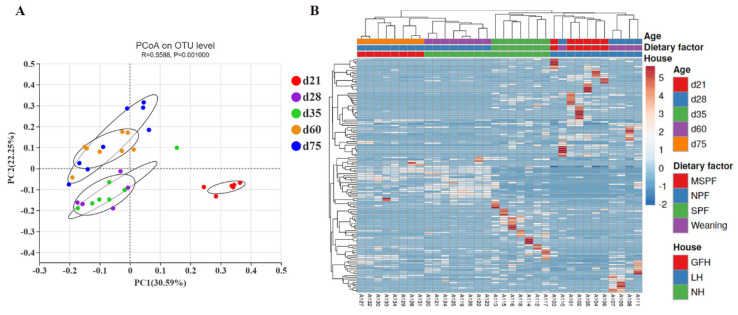
The β diversity of the microbial community expressed as PCoA (**A**) and Heatmap (**B**) at OTU level.

The β-diversity of microbial communities in all samples were analyzed and presented with PCoA (*p <* 0.01, Figure 2A). The first two components accounted for 52.84% variation and great variation were observed among different ages. Similar microbial community characteristics were shown between d 28 and d 35, and between d 60 and d 75. Intriguingly, microbial communities at d 21 were significantly different from the microbiota of other ages. The relative abundance of top 200 OTUs was calculated and drawn into a heatmap after calculating β-diversity in order to further reflect the driving factors of the evolution of microbial communities among different ages (Figure 2B).

### 3.2. Structural Changes of the Microbial Community

**Figure 3:**
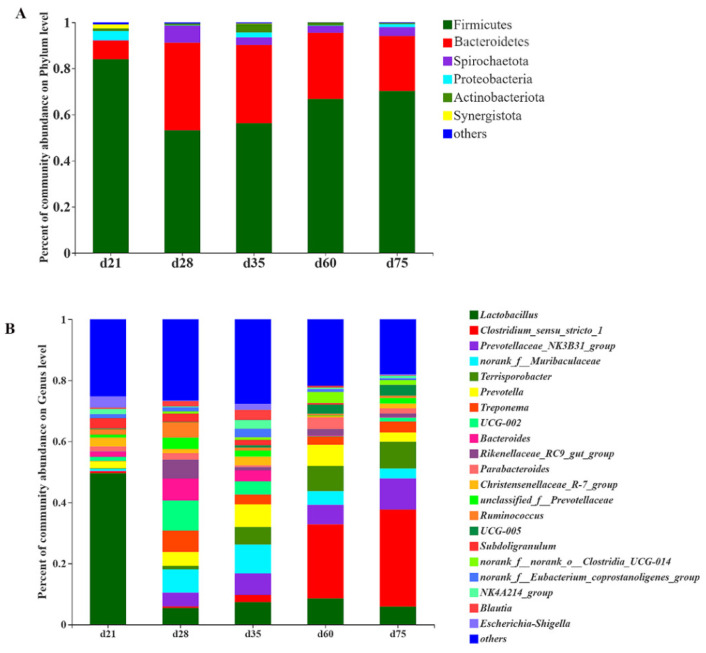
The microbial composition at the phylum level (**A**) and the family level (**B**) (at least one sample relative abundance ≥ 0.1%).

At the phylum level, Firmicutes and Bacteroidetes were the dominant bacteria and their relative abundance accounted for about 90% (Figure 3A). The relative abundance of Firmicutes and Proteobacteria of the samples from d 21 were higher than those from the older ages (Figure 3A), while Bacteroides and Spirochetes showed highest relative abundances on d 28 (Figure 3A). Moreover, Bacteroides showed a trend of decreased relative abundance whereas Firmicutes showed a trend of increased relative abundance after 28 days of age (Figure 3A). At the genus level, *Lactobacillus*, with an average relative abundance close to 50%, dominates the samples from d 21 (Figure 3B). However, the relative abundance of *Lactobacillus* in samples from other ages was significantly lower than those from d 21 (Figure 3B). In the samples from d 60 and d 75, *Clostridium_sensu_stricto_1* was the dominator of microbiota with an average relative abundance of more than 20% (Figure 3B). In addition, *Prevotella* and *Terrisporobacter* had a significantly lower relative abundance in the samples from d 21 than those from older ages (Figure 3B).

**Figure 4:**
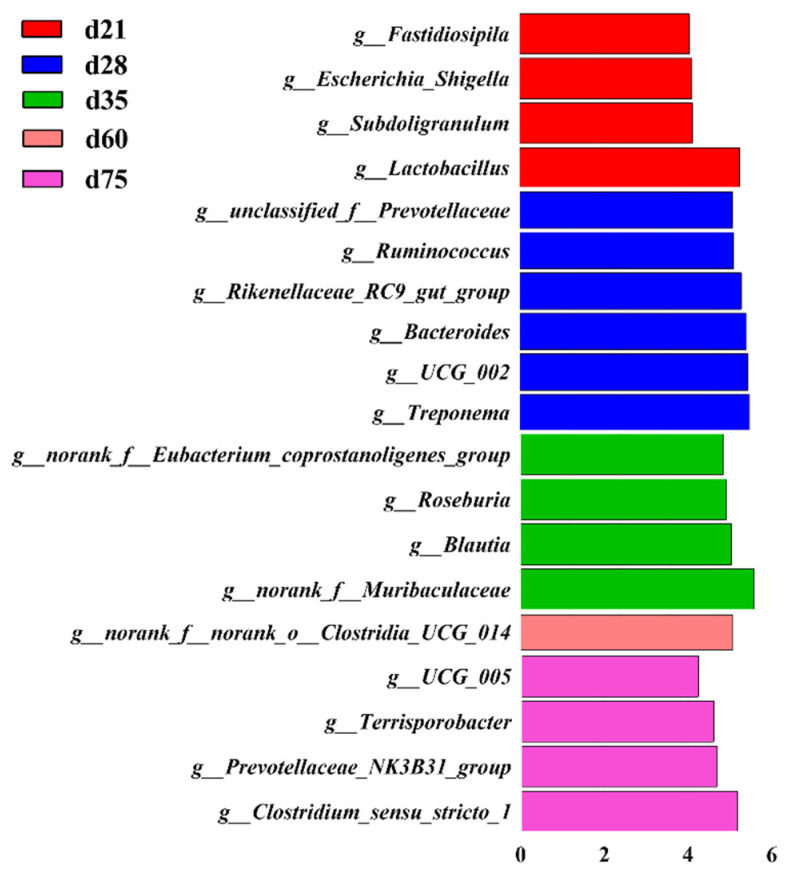
Identification of the most differentially abundant microbes among samples from 5 stages. The genera enriched in the samples among different stages. The plots were generated from Linear Discriminant Analysis Effect Size (LEfSe) analysis with CSS-normalized OTU table and displays taxa with LDA scores above 4.0 and *p*-values below 0.05.

A Lefse analysis was performed to screen the microbes with the most representative characteristics of each age from phylum level to genus level. The results showed that a total of 63 OTUs were identified with an average LDA score greater than 4.00, wherein 21, 18, 12, 3, and 9 OTUs were identified in the samples from d 21, d 28, d 35, d 60 and d 75, respectively (Figure S2). At genus level, the cladogram plot showed high relative abundance of *Lactobacillus*, *Subdoligranulum*, *Escherichia_Shigella* and *Fastidiosipila* in fecal samples of pigs from d 21, *Treponema*, *UCG_002*, *Bacteroides*, *Rikenellaceae_RC9_gut_group*, *Ruminococcus* and *unclassified_f__Prevotellaceae* in fecal samples of pigs from d 28, *norank_f__Muribaculaceae*, *norank_f__Eubacterium_coprostanoligenes_group*, *Roseburia*, *Blautia* in d 35, *norank_f__norank_o__Clostridia_UCG−014* in d 60 and *Clostridium_sensu_stricto_1*, *Prevotellaceae_NK3B31_group*, *Terrisporobacter* and *UCG_005* in d 75, respectively (Figure 4).

### 3.3. Fermentation Characteristics Changes of the Microbial Community

**Figure 5:**
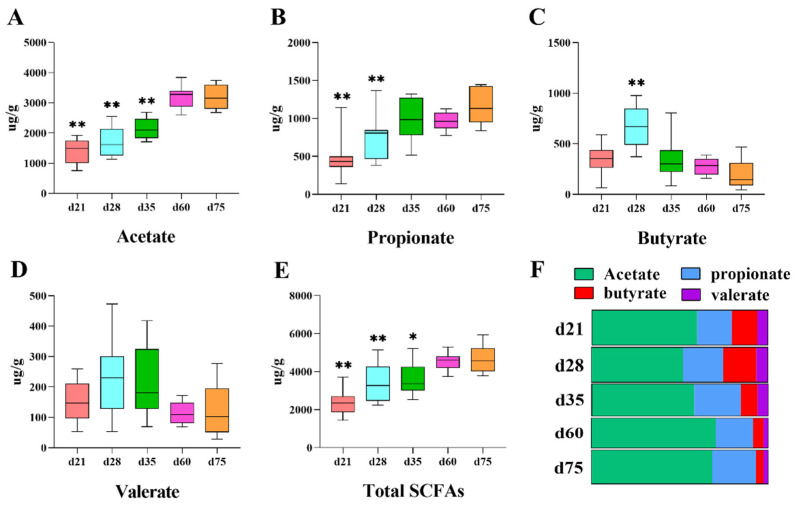
The SCFAs composition in fecal samples from different stages, acetate (**A**), propionate (**B**), butyrate (**C**), valerate (**D**), total SCFAs (**E**), the relative content of each SCFA in different stages, obtained by dividing a single SCFA per sample by the total SCFA (**F**). “*” and “**” in the box-plots represent significant difference compared with the samples from d75, *p <* 0.05 and *p <* 0.01, respectively. The same below.

As shown in Figure 5, fecal SCFAs were used to reflect the fermentation characteristics of gut microflora for NXP at different ages. The concentration of fecal SCFAs between d 60 and d 75 was not significantly different (Figure 5). Compared to d 75, NXP at d 21 and d 28 showed lower concentrations of acetate, propionate and total SCFAs, while NXP at d 35 had lower concentrations of acetate and total SCFAs (*p* < 0.01) (Figure 5A,B,E). However, the fecal samples of NXP at d 28 had a significantly higher butyrate concentration than from d 75 (*p* < 0.01) (Figure 5C). The ratio of acetate, propionate, butyrate and valerate in total SCFAs was calculated and shown in Figure 5F, suggesting that NXP at d 60 and d 75 had higher proportion of acetate and lower proportion of butyrate in fecal samples (*p* < 0.05) compared to d 21 or d 28.

### 3.4. Correlation of Bacteria and SCFAs

**Figure 6:**
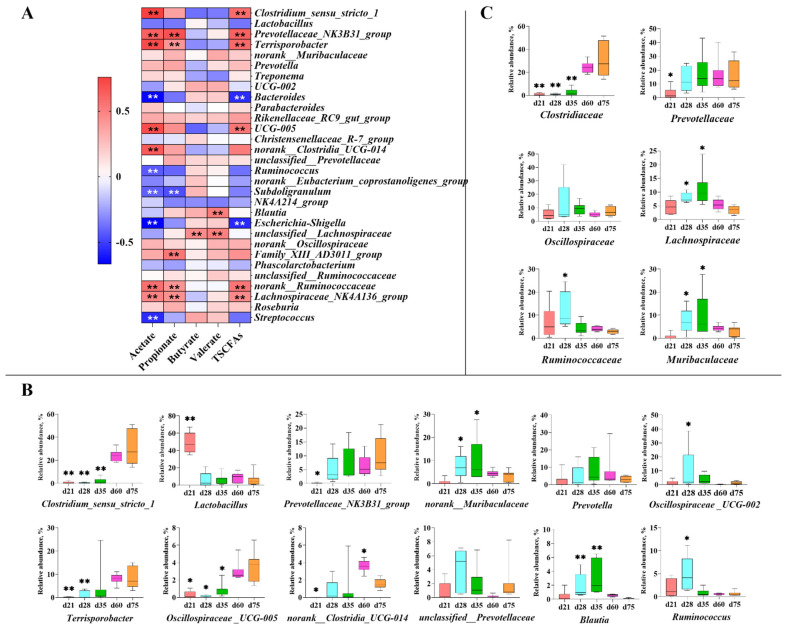
The correlation between the top 30 genera and the concentration of SCFAs (**A**); Main fiber-degrading bacteria at the genus level (**B**) and family level (**C**). “*” and “**” represent significant difference, *p <* 0.05 and *p <* 0.01, respectively.

As shown in Figure 6, The correlation between the top 30 genera and the concentration of SCFAs in feces has been shown in Figure 6A, wherein the bacteria including *Clostridium_sensu_stricto_1*, *Prevotellaceae_NK3B31_group*, *Terrisporobacter*, *Bacteroides*, *Oscillospiraceae_UCG−005*, *norank__Clostridia_UCG−014*, *Ruminococcus*, *Subdoligranulum*, *Escherichia-Shigella*, *norank_Ruminococcaceae*, *Lachnospiraceae_NK4A136_group*, and *Streptococcus* had a strong correlation with the concentration of total SCFAs (*p* < 0.01). In addition, the bacteria including *Clostridium_sensu_stricto_1*, *Prevotellaceae_NK3B31_group*, *Terrisporobacter*, *Bacteroides*, *Oscillospiraceae_UCG−005*, *Escherichia-Shigella*, *norank_Ruminococcaceae*, and *Lachnospiraceae_NK4A136_group* had a strong correlation with the concentration of acetate (*p* < 0.01). In addition, the relative abundance of main fiber-degrading bacteria was compared to reflect the effects of dietary factors at different ages in microbiome at the genus and family levels, respectively (Figure 6B,C). *Lactobacillus* dominated on d 21 whereas the relative abundance of *Prevotellaceae* (mainly *Prevotellaceae_NK3B31_group*), *Lachnospiraceae* (mainly *Blautia*), *Ruminococcaceae* (mainly *Ruminococcus*), *Muribaculaceae* (mainly a norank_ *Muribaculaceae* genus), and one genus, *UCG−002* from *Oscillospiraceae*, increased from d 21 to d 28. In addition, the relative abundance of *Lachnospiraceae* and *Muribaculaceae* decreased after d 35, whereas the relative abundance of *Ruminococcaceae* and *UCG−002* decreased after d 28. The last, three genera including *Clostridium_sensu_stricto_1*, *Terrisporobacter* and *Oscillospiraceae_UCG−005*, enriched in the samples from d 60 and d 75.

## 4. Discussion

The current study evaluated the evolution of intestinal microbiota of NXP in youth. The previous study suggested that microbial colonization was a process from simple to complex accompanying the intestinal development [3,4,5]. In the early life of mammals, the colonized microbes in the gut mainly come from their mother and the environment, while many external microbes cannot be colonized in the gut for a long time [3,4,5]. Our results showed that the total number of OTUs increased from d 21 to d 75; the significant increase in the ACE index was found from d 21 to d 60. ACE index reflects the number of OTUs in the community. The increased ACE index in the current study may be due to two factors. On the one hand, with the change of diets, some bacteria with very low abundance proliferated, and on the other hand, more environmental microbes come into the community [3,4]. Meanwhile, a total of 294 OTUs coexisted in fecal samples from different ages, which may be the core microbes in the intestinal tract of NXP. A rapid change in the diversity index of the microbial community happened between d 21 and d 28, which may be caused by weaning [7,16].

Our results also proved that diet was the main driving factor for the evolution of intestinal microbiota of NXP, agreeing with previous studies [4,7,16]. In the PCoA analysis, the same diet caused similar principal component characteristics between d 60 and d 75, d 28 and d 35, respectively. However, the characteristics of d 28 were closer to those of d 21 rather than d 35 according to the results of heatmap, indicating that the evolution of intestinal microbiota of NXP was nonlinear or incoherent with time before and after weaning due to the commercial production. Early weaning caused drastic and unnatural changes in the microbial community because the intestine did not adapt to a completely solid and non-breast-milk diet, which agreed with the studies on piglets from cultivated breeds [16]. After weaning, the microbial community of fecal samples from d 60 was closer to those from d 75 with the same diet than those from d 35 with the same house. In brief, results mentioned above showed that dietary factors promoted the evolution of the microbial community more powerfully than housing factors in NXP.

Due to the breast milk intake, the microbial community was dominated by *Lactobacillus* in the intestine of suckling piglets and was also previously reported in the Yorkshire piglets [17]. Our results in this experiment showed that *Firmicutes* (relative abundance > 80%), especially *Lactobacillus* (relative abundance about 50%), dominated the intestinal tract of suckling NXP, similar to the report on Yorkshire × Landrace piglets [7]. Interestingly, we found weaning caused a rapid proliferation of *Bacteroides* phylum in the intestine of NXP. Previous studies reported anorexia and fat loss in piglets within a short period of time after weaning [16,18]. The *Firmicutes*/*Bacteroides* (F/B) was positively correlated with the energy storage capacity of pigs, so the decreasing F/B ratio may reflect the negative energy balance of NXP after weaning [19]. In addition, *Spirochaetota*, a phylum containing many pathogenic microorganisms commonly found in pig intestines such as *Treponema*, was also significantly increased in samples from d 28 [20,21]. The increase in *Spirochaetota* may be due to a temporarily decreased immunity of NXP caused by the reduced maternal immunoglobulin intake during weaning [16,22]. Notably, the relative abundance of *Spirochaetota* decreased significantly in the samples of d 35 and subsequent ages, indicating the recovery or improvement of immunity of NXP.

We have identified more than 10 bacteria capable of degrading plant-derived fiber or having strong correlation with SCFAs production among the 19 characteristic genera based on lefse analysis, except *Escherichia_Shigella*, *Rikenellaceae_RC9_gut_group*, *Treponema,* and *Bacteroides* [23,24,25,26,27,28]. Especially in pigs after age of 35 days, almost all characteristic microbes, *Roseburia*, *Blautia*, *norank_f__norank_o__Clostridia_UCG−014*, *Clostridium_sensu_stricto_1*, *Prevotellaceae_NK3B31_group*, *Terrisporobacter* and *UCG_005* were fiber-degrading bacteria at each growing stage [28,29,30,31]. Therefore, the rapid proliferation of fiber-degrading bacteria may be the most important feature of microbial community evolution after weaning, which may enable the intestinal microflora of young NXP to rapidly adapt to plant-derived feeds, especially those containing abundant fiber components [26]. This phenomenon was similar but more obvious than that reported by a previous study in cultivated breed pig with early exposure to plant-based feeds, which may provide a potential explanation for high-fiber feed tolerance in indigenous pig breeds [7,16,32].

The microbes in the large intestine mostly are anaerobic bacteria and could ferment plant cell wall components to produce SCFAs, thus providing energy for pigs [32]. It was reported that about 90% of these SCFAs, in the form of acetate, propionate and butyrate, could be absorbed and may play different roles. For instance, butyrate provides energy to intestinal epithelial cells whereas acetate and propionate enter the fatty acid synthesis and gluconeogenesis pathways, respectively [33]. Unabsorbed SCFAs are excreted with feces and could partly reflect microbial fermentation characteristics [34]. Accordingly, our results showed that the intestinal fermentation capacity of NXP enhanced continuously from d 21 to d 60 with an increasing concentration of total SCFAs. Additionally, the proportion of acetate continued to rise whereas the ratio of butyrate kept decreasing after d 28. In addition, the bacteria that are strongly correlated with the total SCFAs concentration are also strongly correlated with the acetate concentration, possibly because acetate is the main contributor to total SCFAs.

The relative abundance of *Lactobacillus* decreased at weaning and remained a constant level after that, which may be because of the reduced intake of breast milk oligosaccharides, thus losing its dominant position in the intestinal tract of NXP [16,17]. After weaning, some bacteria multiplied including *Prevotellaceae* (mainly *Prevotellaceae_NK3B31_group*), *Lachnospiraceae* (mainly *Blautia)*, *Ruminococcaceae* (mainly *Ruminococcus*), *Muribaculaceae* (mainly a norank_ *Muribaculaceae* genus)*,* and *Oscillospiraceae_UCG−002*. These bacteria have been found to be beneficial for piglets to obtain energy from plant-derived feed [23,24,25,26,27,28,29,30,31]. However, their relative abundance was no longer increased from d 35 to d 75. Moreover, with the exception of *Prevotellaceae*, the relative abundance of these bacteria decreased significantly at d 60 and d 75 compared to d 28 or d 35, and their dominant positions were replaced by *Clostridium_sensu_stricto_1*, *Terrisporobacter* and *Oscillospiraceae*_*UCG−005* with significant correlations with acetate and total SCFAs concentration were observed. *Prevotellaceae* has always maintained its dominant position in the intestines of NXP after weaning, possibly because of its extensive utilization of the fibers derived from plant cell walls and even the complex polysaccharide structure [35,36]. Previous studies reported that mature pigs were more adaptable to high-fiber diets than young pigs; thus, they have higher fiber digestibility, mainly due to differences in intestinal tissue and microbiota [37,38,39]. The dominance of *Clostridium_Sensu_Stricto_1* and *Terrisporobacter* in d 75 NXP was similar to those in the finishing Duroc × Large White × Landrace pigs in a previous study, but their relative abundance in NXP was higher than those in Duroc × Large White × Landrace pigs [32]. *Clostridium_sensu_stricto_1* and *Terrisporobacter* have been reported to play an important role in the in vitro anaerobic fermentation of rice straw, which is difficult to be digested by monogastric animals [40]. However, *UCG−005*, as a bacterium that could not be cultured temporarily, has not been reported. We speculated that the increase in relative abundance of these three bacteria may be beneficial for NXP to adapt to a high-fiber diet and obtain energy from it, but we were not yet able to determine the mechanism. Therefore, subsequent studies need to identify whether there is a relationship between the intestinal development and the microbial evolution in NXP, wherein the role of dietary factors, especially dietary fiber, also needs to be systematically studied.

## 5. Conclusions

In conclusion, the evolution of gut microbiota was mainly adapted to the change of dietary factors during NXP growth. The response of fiber-degrading bacteria at different stages may help NXP better adapt to plant-derived feed. Further studies need to identify the relationship between intestinal development and the microbial evolution, and to determine the role of dietary factors, especially dietary fiber, in this relationship.

## Figures and Tables

**Table 1:** Chemical composition of breast milk and feeds of Ningxiang pigs.

	Breast Milk ^1^	SPF ^1^	NPF ^1^
DM, %	14.05	90.19	89.00
Protein ^2^, %	5.17	16.95	13.60
SDF, %	—	1.58	1.05
IDF, %	—	9.03	14.75
TDF, %	—	10.62	15.80
CF, %	—	2.13	5.07
Fat, %	3.62	5.25	3.59
Lactose, %	4.80	6.03	—
Starch, %	—	32.38	49.91

^1^ The breast milk was collected at d 21; SPF, suckling pig feed; NPF, nursery pig feed. ^2^ The protein in the milk sample was shown as true protein, while the protein in the feeds was shown as crude protein.

## Data Availability

Not available.

## Supplementary Materials

The following are available online at https://www.mdpi.com/2076-2615/11/3/638/s1, Figure S1: Dietary and housing factors experienced by NXP at different ages, Figure S2: The cladogram plot of Lefse Analysis (LDA value > 4.0) from phylum level to genus level. Table S1: Formulations for Suckling pig feed (SPF) and nursery pig feed (NPF), Table S2: Body weight and age of pigs at the time of sample collection, Table S3: The microbial composition at the phylum level, Table S4: The microbial composition at the family level.
