# Supplementary material for: Evolution of the Gut Microbiota and Its Fermentation Characteristics of Ningxiang Pigs at the Young Stage
Source: Animals (Basel). 2021 Feb 27;11(3):638. doi: 10.3390/ani11030638 (PMC7997423; doi:10.3390/ani11030638)

Supplementary Materials

Table S1 Formulations for Suckling pig feed (SPF) and nursery pig feed (NPF).

	SPF	NPF
Corn	33.00	57.00
Extruded corn	11.00	—
Unhusked rice	—	10.00
Broken Rice	10.00	—
Whey powder	8.00	—
Sucrose	2.00	—
Glucose	2.00	—
Soybean oil	1.00	—
Olive oil	2.00	—
Soybean meal	3.00	18.00
Defatted rice bran	—	6.00
Fermented soybean meal	5.00	2.00
Full-fat extruded soybean	9.00	4.00
Fish meal	4.00	—
Plasma protein powder	3.00	—
Mixed small peptide [1]	2.00	—
Yeast hydrolysate	1.50	—
Hydrochloride-Lysine (70% Lys)	0.47	—
Threonine (98% Thr)	0.23	—
Methionine (98% Met)	0.12	—
Tryptophan (98% Try)	0.08	—
Vitamin-mineral premix [2]	2.60	3.00
Total	100.00	100.00

[1] Hydrolyzed from 40% lactoferrin and 60% soybean protein, each peptide molecule contains 2 to 10 amino acids.

[2] SPF was formulated according to NRC 2012. Vitamin and mineral premix provided the following per kilogram of diet, 11000 IU vitamin A as vitamin A acetate, 1500 IU vitamin D as vitamin D3, 45 IU vitamin E as dl-α-tocopheryl acetate, 4 mg vitamin K as menadione sodium bisulfate, 1.1 mg of vitamin B1, 5.0 mg of vitamin B2, 1.8 mg of vitamin B6, 32 μg of vitamin B12, 5.6 mg d-pantothenic acid as calcium pantothenate, 11 mg of nicotinic acid, 165 mg choline as choline chloride, 20 mg Fe as iron sulfate, 30 mg Cu as copper sulfate, 80 mg Zn as Zinc sulfate, 20 mg Mn as manganese oxide, 0.3 mg I as ethylenediamine dihydroiodide, and 0.2 mg Se as sodium selenite

NPF was formulated according to NRC 2012. Vitamin and mineral premix provided the following per kilogram of diet, 6,500 IU vitamin A as vitamin A acetate, 2,500 IU vitamin D as vitamin D3, 22.0 IU vitamin E as dl-α-tocopheryl acetate, 2.1 mg vitamin K as menadione sodium bisulfate, 0.98 mg of vitamin B1, 5.3 mg of vitamin B2, 2 mg of vitamin B6, 11.8 μg of vitamin B12, 11.3 mg d-pantothenic acid as calcium pantothenate, 22 mg of nicotinic acid, 400 mg choline as choline chloride, 0.4 mg of folic acid, 40 μg of biotin, 120 mg Fe as iron sulfate, 125 mg Cu as copper sulfate, 20 mg Mn as manganese oxide, 0.40 mg I as ethylenediamine dihydroiodide, and 0.30 mg Se as sodium selenite.

Table S2 Body weight and age of pigs at the time of sample collection.

Piglet id	Body weight/kg					Sample id				
	d21	d28	d35	d60	d75	d21	d28	d35	d60	d75
NXP1	5.28	7.01	8.97	17.72	23.57	A101	A107	A112	A119	A127
NXP2	4.78	6.07	7.82	15.45	20.84	NA[1]	A108	NA[1]	A120	A128
NXP3	5.13	6.75	8.63	17.26	22.95	A102	A109	A113	A121	A129
NXP4	4.92	6.53	8.34	16.75	22.15	A103	NA[1]	A114	A122	A130
NXP5	5.24	6.85	8.80	17.51	23.30	A104	A110	A115	A123	A131
NXP6	5.04	6.66	8.56	16.87	22.52	A105	NA[1]	A116	A124	A132
NXP7	4.98	6.54	8.41	16.79	22.33	A106	A111	A117	A125	A133
NXP8	4.81	6.32	7.99	16.20	21.67	NA[1]	NA[1]	A118	A126	A134

[1] NA: Samples were not used for microbial analysis due to contamination or low extraction quality of DNA.

Table S3. The microbial composition at the phylum level (at least one sample relative abundance ≥ 0.1%).

	d 21	d 28	d 35	d 60	d 75	SEM	*P*-value
Firmicutes	84.375	52.780	54.618	66.629	70.139	2.762	0.001
Bacteroidetes	7.878	37.607	35.856	28.891	23.853	2.620	0.002
Firmicutes/Bacteroidetes	14.614	1.678	2.768	2.523	4.237	1.140	0.001
Spirochaetota	0.140	8.101	3.303	2.961	3.929	0.840	0.107
Actinobacteriota	1.179	0.795	3.266	1.271	0.570	0.282	0.010
Proteobacteria	3.760	0.212	2.190	0.168	1.297	0.460	0.084
Synergistota	1.774	0.013	0.282	0.002	0.003	0.200	0.019
others	0.895	0.491	0.486	0.078	0.209	0.082	0.010

Table S4. The microbial composition at the family level (at least one sample relative abundance ≥ 0.1%).

	d 21	d 28	d 35	d 60	d 75	SEM	*P*-value
Clostridiaceae	0.889	1.069	2.779	24.592	31.524	2.662	0.000
Prevotellaceae	3.125	13.583	19.010	16.549	16.346	1.891	0.078
Lactobacillaceae	48.946	5.885	7.386	8.679	6.264	3.067	0.000
Oscillospiraceae	5.242	12.324	9.411	5.043	7.482	1.206	0.361
Lachnospiraceae	4.698	8.012	11.483	5.535	3.493	0.730	0.001
Peptostreptococcaceae	0.201	1.249	4.769	8.756	8.832	1.014	0.007
Ruminococcaceae	6.904	12.220	4.228	4.018	2.945	0.894	0.011
Muribaculaceae	0.656	7.517	10.002	4.461	3.279	0.963	0.017
Spirochaetaceae	0.140	8.101	3.303	2.961	3.929	0.840	0.107
Rikenellaceae	0.527	6.208	1.345	2.203	1.276	0.516	0.009
Bacteroidaceae	1.728	6.900	3.996	0.231	0.157	0.991	0.203
Tannerellaceae	1.671	2.084	0.605	3.777	1.681	0.455	0.220
Christensenellaceae	3.453	1.432	2.741	0.460	1.672	0.458	0.267
norank_o__Clostridia_UCG-014	0.002	0.733	0.968	3.619	1.581	0.291	0.000
Erysipelotrichaceae	1.483	2.598	2.239	0.442	1.210	0.238	0.027
Eubacterium_coprostanoligenes_group	1.308	1.342	2.930	0.983	0.640	0.276	0.057
Anaerovoracaceae	2.353	1.991	1.425	0.838	0.912	0.221	0.130
Enterobacteriaceae	3.406	0.177	2.057	0.003	0.345	0.436	0.055
Acidaminococcaceae	2.802	0.538	1.269	0.772	0.133	0.257	0.008
Streptococcaceae	2.291	1.462	0.208	0.023	0.045	0.400	0.302
norank_o__RF39	0.001	0.673	0.447	1.104	0.355	0.100	0.003
Coriobacteriaceae	0.295	0.307	1.457	0.424	0.109	0.167	0.061
Atopobiaceae	0.347	0.070	1.239	0.264	0.126	0.138	0.035
Synergistaceae	1.774	0.013	0.282	0.002	0.003	0.200	0.019
Hungateiclostridiaceae	2.314	0.000	0.000	0.006	0.002	0.350	0.173
Veillonellaceae	0.322	0.151	0.851	0.000	0.000	0.089	0.003
Carnobacteriaceae	0.000	0.000	0.000	0.207	0.723	0.122	0.220
Moraxellaceae	0.000	0.000	0.021	0.082	0.765	0.105	0.050
norank_o__Bacteroidales	0.008	0.795	0.120	0.009	0.001	0.079	0.009
others	3.115	2.568	3.429	3.957	4.170	0.222	0.177

Figure S1. Dietary and housing factors experienced by NXP at different ages.

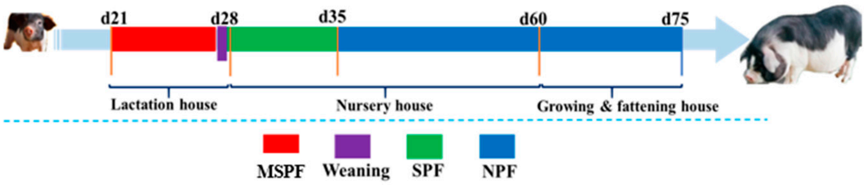

Figure S2. The cladogram plot of Lefse Analysis (LDA value > 4.0) from phylum level to genus level.

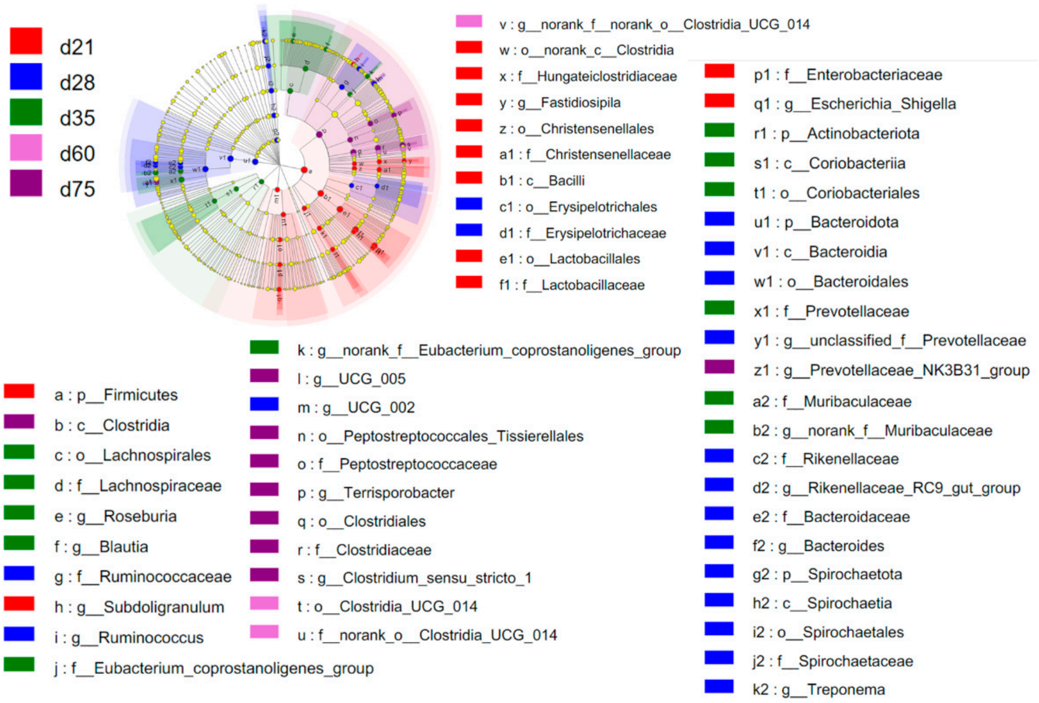

Supplement: Supplementary file 1 [file animals-11-00638-s001.pdf]
